# SARS-COV-2 causes significant abnormalities in the fibrinolysis system of patients: correlation between viral mutations, variants and thrombosis

**DOI:** 10.3389/fcimb.2025.1531412

**Published:** 2025-04-15

**Authors:** Esra’a Abudouleh, Tarek Owaidah, Fatimah Alhamlan, Arwa A. Al-Qahtani, Reem M. Aljowaie, Fatimah Al-Ghnnam, Marie Fe Bohol, Ahmed A. Al-Qahtani

**Affiliations:** ^1^ Department of Botany and Microbiology, College of Science, King Saud University, Riyadh, Saudi Arabia; ^2^ Department of Pathology and Laboratory Medicine, King Faisal Specialist Hospital and Research Center, Riyadh, Saudi Arabia; ^3^ Department of Pathology, College of Medicine, Alfaisal University, Riyadh, Saudi Arabia; ^4^ Department of Infection and Immunity, Research Centre, King Faisal Specialist Hospital & Research Centre, Riyadh, Saudi Arabia; ^5^ Department of Microbiology and Immunology, College of Medicine, Alfaisal University, Riyadh, Saudi Arabia; ^6^ Department of Family Medicine, College of Medicine, Al-Imam Mohammad Ibn Saud Islamic University, Riyadh, Saudi Arabia

**Keywords:** SARS-CoV2 mutations, SARS-CoV-2 RNAemia, thrombosis, thrombin, plasminogen activator inhibitor (PAI-1), tissue plasminogen activator (tPA), activatable fibrinolysis inhibitor

## Abstract

**Background:**

Coronavirus disease (COVID-19) is reported as a complex disorder affecting multiple systems and coagulopathy that can cause mortality. In this study, we investigated the correlation of SARS-CoV-2 mutations found in blood samples with various changes in the fibrinolysis system, as well as the severity of the disease based on outcome and whether or not these patients were admitted into the ICU.

**Materials and methods:**

COVID-19 patients (n = 446) admitted to our institute between 2021 and 2022 were recruited. Blood samples were collected, and a sequence analysis of the SARS-CoV-2 spike gene was isolated from the blood. Measured several parameters of fibrinolysis and coagulation, including alpha-2-antiplasmin and plasminogen, thrombin activatable fibrinolysis inhibitor (TAFI), tissue plasminogen activator (tPA), plasminogen activator inhibitor-1 (PAI-1), D-dimer, and fibrinogen levels.

**Results:**

SARS-CoV-2 RNA was found in 123/446 (27.6%) of the blood samples. The N501Y, D614G, K417N, and P681R mutations among COVID-19 patients were associated with higher admissions to the ICU (P = 0.0057, P = 0.0068, P = 0.0193, and P = 0.018, respectively). Omicron (BA.1.1) variant variants are highly associated with thrombosis (P = 0.002) in hospitalized COVID-19 patients that are unvaccinated and have comorbidity conditions. The plasma levels of tPA, aPTT, and D-dimer were significantly higher in participants who had the N501Y mutation (P = 0.044, P = 0.024, and P = 0.027, respectively).

**Conclusion:**

Thrombosis was the most prevalent condition among severe COVID-19 patients. The correlation between specific SARS-CoV-2 new variants and thrombosis warrants more investigation.

## Introduction

Severe acute respiratory syndrome coronavirus 2 (SARS-CoV-2) is the pathogen responsible for coronavirus disease-19 (COVID-19), a highly contagious respiratory disease. COVID-19 manifests as a multisystem end-organ disorder, which may be triggered by thromboinflammation disorder that affects several organ systems. SARS-CoV-2 systematically disseminate through the blood or lymphatics (possibly involving direct lymphocyte infection) and is mainly passed on by localized pulmonary infection (Li et al., 2020).

The impact of SARS-CoV-2 on the coagulation system has been extensively studied since the onset of the pandemic. It is well-documented that severe COVID-19 cases exhibit significant abnormalities in coagulation parameters, often leading to a hypercoagulable state. Studies have consistently shown an increased risk of thrombotic events among hospitalized COVID-19 patients, particularly those requiring intensive care (Hadid et al., 2021; Kalbhenn et al., 2021).

This increased clotting tendency can lead to thrombosis, a condition where blood clots form within the blood vessels, leading to serious complications such as pulmonary embolism and stroke (Chung et al., 2015). One of the key mechanisms involved in the progression of respiratory diseases is the activation of fibrinolysis. In these diseases, there is an increase in the release of fibrinolytic enzymes, such as plasmin, that breakdown blood clots formed in the lung (Hassanpour et al., 2020). This excessive fibrinolysis can contribute to the progression of the disease by causing an increase in the permeability of the lung vessels, leading to fluid accumulation in the lungs and a further reduction in oxygen exchange (Edlmann et al., 2017).

Several coagulation and fibrinolytic factors, including plasminogen, alpha-2-antiplasmin(α2AP), tissue plasminogen activator (tPA), fibrinogen, thrombin activated fibrinolysis inhibitor (TAFI), plasminogen activator inhibitor-1 (PAI-1), and D-dimer, have been found to be involved in the pathogenesis of COVID-19-related coagulopathy (Henry et al., 2021).

Mutations in the SARS-CoV-2 Spike protein (SP) have been linked to varying degrees of disease severity and coagulopathy. For instance, the N501Y, D614G, K417N, and E484K mutations have been associated with higher Intensive Care Unit (ICU) admissions and increased mortality rates. The Omicron (BA.1.1) variant, characterized by the N501Y mutation, has shown a significant correlation with thrombosis in unvaccinated patients with comorbid conditions. Increased plasma levels of fibrinolytic and coagulation markers, such as tPA, aPTT, and D-dimer, have been reported in patients with certain SARS-CoV-2 mutations. These elevated levels are indicative of hyperfibrinolysis and coagulopathy, contributing to the severe clinical manifestations observed in COVID-19 patients. Specifically, the N501Y mutation has been strongly associated with elevated plasma levels of tPA, aPTT, and D-dimer, highlighting its potential role in the pathogenesis of COVID-19-related thrombosis (Hadid et al., 2021; Kalbhenn et al., 2021).

Despite the extensive research, there remain gaps in our understanding of the exact mechanisms through which specific SARS-CoV-2 variants influence the coagulation system. Further studies are needed to elucidate the thrombogenic potential of different variants and to compare the effects of these variants on various coagulation markers. This would aid in better risk assessment and management of COVID-19 patients.

The aim of this study was to detect the presence of the SARS-CoV2 virus in the blood among patients admitted to the hospital with confirmed COVID-19 using (RT-PCR). Additionally, we aimed to investigate the association between genetic profiles, SARS-CoV-2 variants detected in the blood samples, alterations in the fibrinolysis system, and the occurrence of thrombosis. Furthermore, we assessed the severity of the disease based on outcome, disease stages, and whether these patients were admitted to the ICU. Our findings have the potential to inform the development of effective therapeutic strategies targeting the disease process. For example, the identification of specific SARS-CoV-2 mutations, such as N501Y and D614G, which are associated with increased thrombosis risk, could lead to the development of targeted anticoagulant therapies. These therapies could be tailored to patients with these mutations to prevent thrombotic complications and improve clinical outcomes.

## Materials and methods

### Study design and participants

COVID-19 patients (n = 446) admitted to our institute between February 21, 2021, and March 17, 2022 were recruited for this study. Patients were identified and selected based on their admission records to the internal medicine department and the ICU. Inclusion criteria included a confirmed diagnosis of COVID-19 through PCR testing, and patients ranged in age from 8 days to 96 years. Informed consent was obtained from all participants or their legal guardians before enrollment. The Ethics Committee of KFSH&RC approved this study (IRB No.: 2201086), and the study was conducted in accordance with the Declaration of Helsinki, 1975. Patients were recruited from both the internal medicine department (n = 237) (53.14%) and the ICU (n = 209) (46.86%). Patients ranged in age from 8 days to 96 years (median: 54.9 years). Just over half the sample (50.45%) was male. A confirmed diagnosis of SARS-CoV-2 was performed by RNA detection of the virus using RT-PCR on both blood samples and combined nasopharyngeal and oropharyngeal swab samples. This comprehensive molecular diagnosis approach ensured accurate detection of SARS-CoV-2 RNA across different specimen types. The study excluded individuals with unconfirmed COVID-19 diagnoses.

The patient’s demographics, clinical data, clinical presentation, and routine laboratory results were obtained by accessing the patient’s electronic medical file on the day of enrollment, the third, the seventh, and the fourteenth. In accordance with hospital policies and the COVID-19 procedure, all patients were clinically categorized as belonging to four separate stages can be used to classify the severity of infection (Abudouleh et al., 2023).

These stages were defined as follows:

- Stage A: Mild symptoms, not requiring hospitalization.
- Stage B: Moderate symptoms, requiring hospitalization but not intensive care.
- Stage C: Severe symptoms, requiring intensive care but not mechanical ventilation.
- Stage D: Critical symptoms, requiring mechanical ventilation and intensive care.

**Figure 1:**
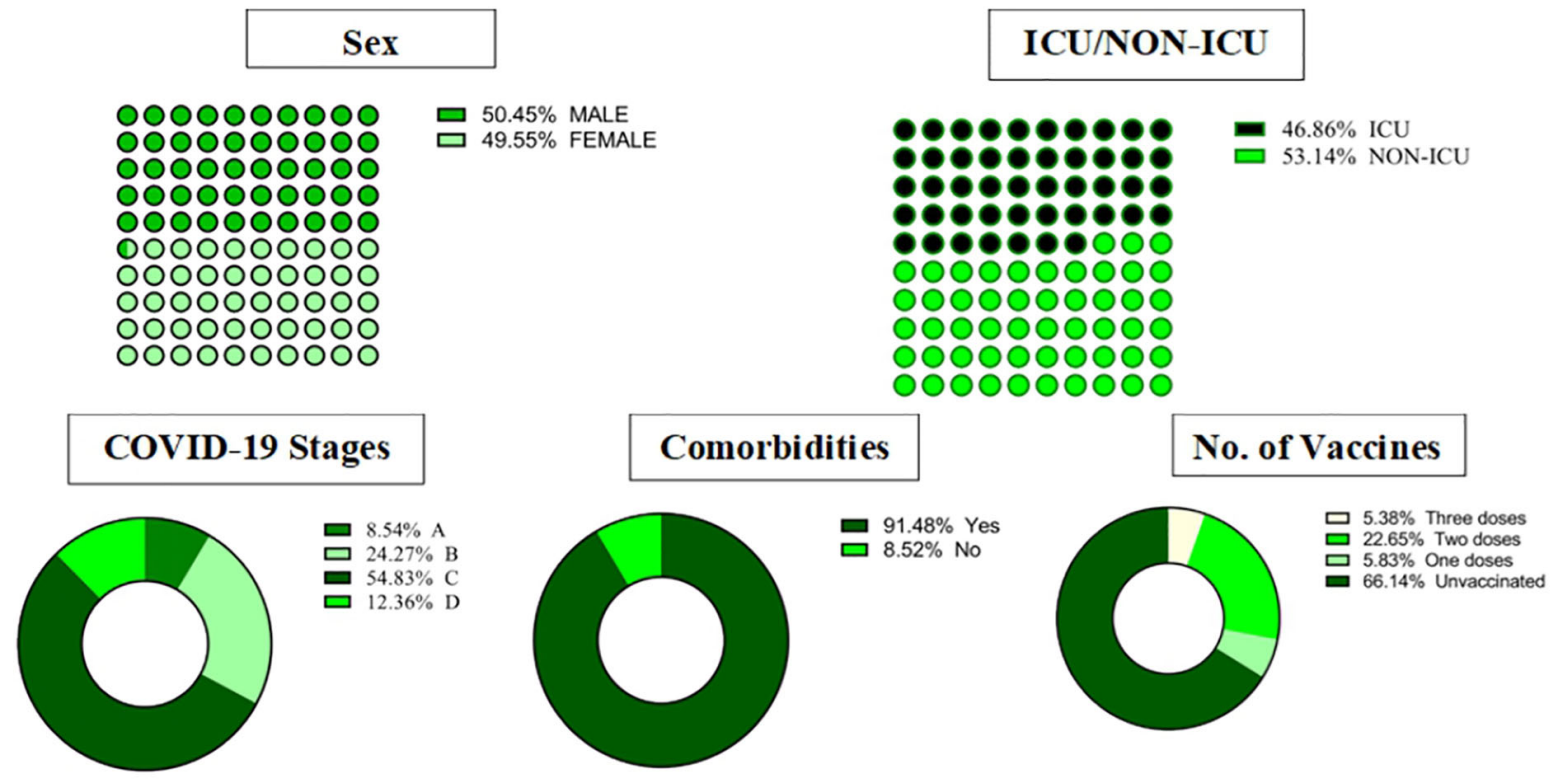
Demographics, clinical characteristics, and outcomes in COVID-19 patients. ICU, intensive care unit.

Stage A was the least severe, while Stage D was the most severe. The distribution of COVID-19 stages among patients was as follows: Stage C was the most common, with 244 cases (54. 83%). %), followed by Stage B with 108 cases (24.27%). Stage D had 55 cases (12.36%). and Stage A was the least frequent, with 39 cases (8.54%). Approximately 90% of the COVID-19 patients exhibited comorbidity conditions, and over 60% of them were unvaccinated (**Figure 1**).

### Laboratory analysis

Blood samples were collected into tubes containing citrated blood (3.2%) and EDTA. When the samples were collected, they were all citrated, centrifuged for 15 minutes at 2000–2500 RCF, and then separated into aliquots to test for various coagulation markers (D-dimer, fibrinogen, prothrombin time, international normalized ratio (INR), and activated partial thromboplastin time (aPTT) at the time of hospital admission using STAR Max^®^ (Diagnostica Stago, Marseille, France). Automated SYSMEX XN-10 (Sysmex Corporation, Kobe, Japan) equipment was used to count the platelets in the EDTA samples. The remaining aliquots were promptly preserved at a temperature of -80°C until the testing process was done.

The plasma concentrations of PAI-1 (Asserachrome #00949), activated and inactivated TAFI (Asserachrome #00616), and tPA (Asserachrome #00948) were assessed using an enzyme-linked immunosorbent assay (ELISA) with colorimetric output, in accordance with the manufacturer’s protocol (Asserachrome, Diagnostica Stago, France). These assays were performed on an ELISA plate reader (SYNERGY-HTX, BioTek Instruments, USA).Furthermore, the plasma concentrations of plasminogen (Stachrom #00658) and α2AP (Stachrom #00659) levels were determined using a colorimetric (STA-Stachrom) assay, employing the synthetic chromogenic substrate method. The measurements were conducted on the STA Compact Max3 Analyzer, a fully automated clinical analyzer from Diagnostica Stago Company (France).

To detect SARS-CoV-2 in blood samples, we performed a series of steps to identify RNAemia and mutations in COVID-19 patients. RNA extraction was conducted on each patient’s blood sample, followed by screening tests on all 446 samples to detect SARS-CoV-2 RNAemia. The extraction was carried out using the MagMAX™ Viral/Pathogen II (MVP II) Nucleic Acid Isolation Kit (Catalog # A42352, Thermo Fisher Scientific, MA, USA) with the KingFisher™ Flex system (Catalog # 5400610, Thermo Fisher Scientific). The quality and quantity of the extracted RNA were assessed using a NanoDrop spectrophotometer, and nucleic acid purity was determined by calculating the absorbance ratio at 260/280 nm, with a ratio of approximately 2.0 indicating pure RNA.

SARS-CoV-2 RNAemia by RT-qPCR with viral load estimated based on CT values of the N, ORF1ab, and S genes. In-house primers, which were previously described (Alhamlan et al., 2023), were used for RNA detection. The detailed RT-qPCR protocol, including primer sequences and reaction conditions, has been outlined in our previous work (Alhamlan et al., 2023).

Whole-genome sequencing was performed for all SARS-CoV-2 RNAemia samples using the Ion AmpliSeq SARS-CoV-2 Research Panel on the Ion Torrent S5™ platform. Variants were identified using the S5 Torrent Suite™ software Variant Caller, version 5.12.

### Statistical analysis

In this investigation, we utilized GraphPad Prism Version 9 for statistical analysis. When comparing two groups, we examined the Mann-Whitney U test for skewed data and a two-sided t-test for normally distributed data. The Shapiro-Wilk test was used to determine normality. Additionally, Fisher’s exact test was performed to contingency tables. Descriptive analysis was applied using bar graphs and dot plots to assess the distribution of instances over time within each group. Significance was defined as a p-value less than 0.05, with two tails. The plasma levels of the examined biomarkers were presented as population medians, accompanied by either the 95% confidence interval (CI) or the interquartile range (IQR).

## Results

### Laboratory results

In patients admitted to the ICU, the white blood cell count, particularly neutrophils, was significantly elevated, while lymphocyte and monocyte counts were notably lower. Our study revealed that ICU patients had markedly higher levels of fibrinogen and D-dimer compared to non-ICU patients. Specifically, the median fibrinogen level was 5.04 g/L (IQR: 0.65-9.71 g/L, P < 0.001), and the median D-dimer level was 1.75 µg/ml (IQR: 0.5 to >20 µg/ml, P = 0.015). In contrast, hemoglobin levels, aPTT, platelet count, and INR did not show significant differences between ICU and non-ICU patients. **Table 1** provides a detailed summary of the selected laboratory results for COVID-19 patients.

**Table 1:** Comparison of hematological and coagulation parameters in ICU and non-ICU COVID-19 patients.

	References range	All (n=446)		ICU (n=209)		Non-ICU (n=237)		P Value
		Median	IQR	Median	IQR	Median	IQR	
**White blood cell (10^9^/L)**	4-11	5.94	0.38-192	6.36	0.46-191.6	5.57	0.38-23.48	**0.012**
**Neutrophil (10^9^/L)**	2-7.5	4.14	0.02-90.08	4.62	0.53-28.68	3.56	0.02-90.80	**<.001**
**Lymphocytes (10^9^/L)**	1.5-4	0.94	0-216	0.81	0-216.4	1.06	0-6.95	**0.002**
**Monocytes (10^9^/L)**	0.20-0.80	0.455	0-4.55	0.38	0-4.55	0.49	0-2.55	**<.001**
**Platelets counts(10^9/^L)**	150-450	207	9-878	209	13-878	202	9-827	0.818
**Hemoglobin(g/L)**	118-148	116	8.22-179	116	8.22-179	117	10-167	0.848
**D-dimer (µg/ml)**	>0.5	1.16	0.27->20	1.75	0.5->20	1.15	0.27->20	**0.015**
**Fibrinogen (g/L)**	1.4-4.40	4.8	0.65-9.71	5.04	0.65-9.71	4.5	0.8-8.730	**<.001**
**PT(Sec)**	10_14	14.9	11.6-72.6	14.9	11.60-17.38	15.1	11.80-16.90	0.498
**aPTT (Sec)**	26-40	38.75	26.3-150	38.75	26.3-150	38.75	26.30-109.6	0.597
**INR**	0.8–1.10	1.1	0.2–21	1.1	0.8–4.3	1.1	0.90–21	0.354

INR, International Normalized Ratio; PT, Prothrombin Time; aPTT, Partial Thromboplastin Time; ICU, Intensive Care Unit; and IQR, Interquartile Range.

On the same day that plasma samples were taken, laboratory tests were performed. Significant differences (P<0.05) are in bold.

### Molecular virology results

**Figure 2:**
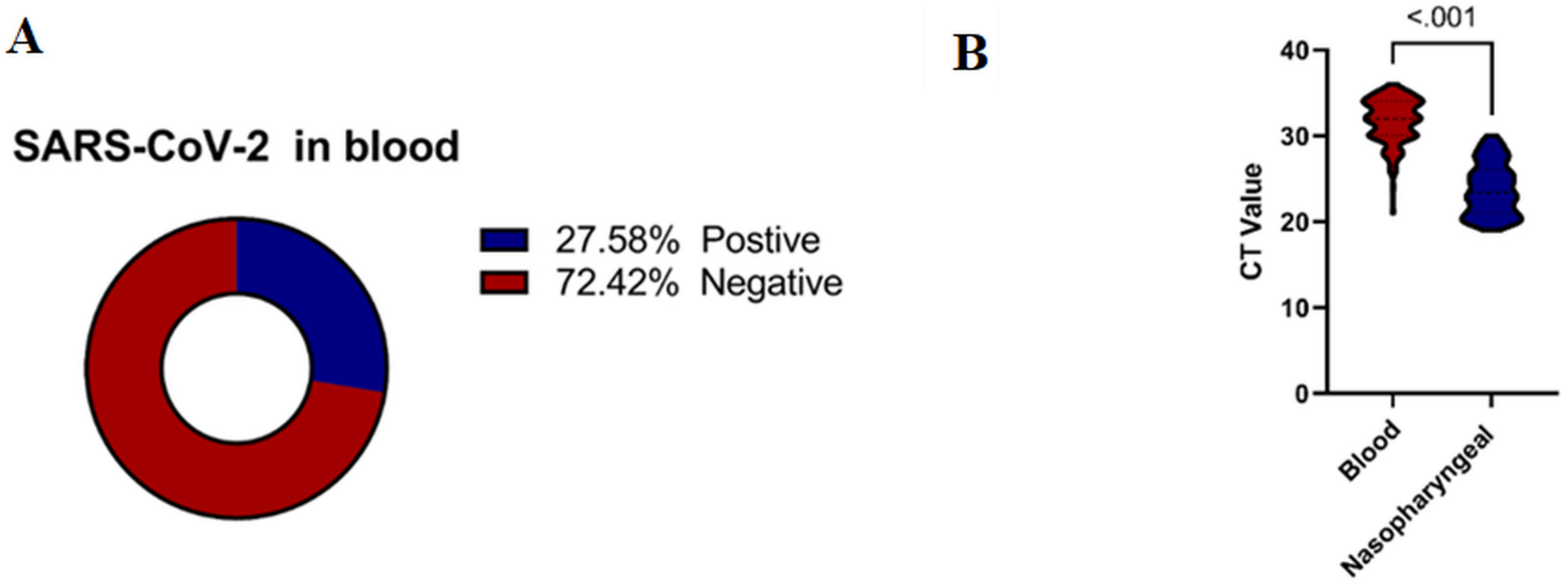
**(A)** Percentage of SARS-CoV-2 RNAemia among COVID-19 Patients. **(B)** Correlation between CT Values in Blood and Nasopharyngeal Samples among COVID-19 Patients. Mann-Whitney U Test was performed for non-normally distributed data, and a two-sided- t-test for normally distributed data. Significant differences are indicated by *P*<0.05.

Specifically, 74 samples (16.6%) were positive in plasma, 28 samples (6.3%) in whole blood, and 21 samples (4.7%) in both whole blood and plasma **Figure 2A**. The cycle threshold (CT) value of the N, ORF ab1, and S genes in blood samples ranged from 24 to 36, with a median of 32 (**Figure 2B**). In contrast, the CT values in nasopharyngeal samples ranged from 19 to 30, with a median of 23.4. Despite the lower CT values in nasopharyngeal samples, blood samples were chosen for sequencing to investigate the presence of SARS-CoV-2 RNAemia and its correlation with disease severity and thrombosis. Sequencing blood samples allows for the detection of viral RNA in the bloodstream, which is associated with more severe disease outcomes and can provide insights into the systemic nature of the infection. This comprehensive analysis underscores the significance of sampling multiple sites to accurately assess the presence and extent of SARS-CoV-2 RNAemia, which may have implications for understanding the systemic nature of the infection and its impact on clinical outcomes.

There was a significant difference in the CT values between blood and nasopharyngeal samples for the N, ORF, and S genes (P < 0.001). These findings highlight the importance of considering different sample types when evaluating SARS-CoV-2 RNAemia targeted the S gene for this evaluation in COVID-19 patients (**Figure 2B**). This comprehensive analysis underscores the significance of sampling multiple sites to accurately assess the presence and extent of SARS-CoV-2 RNAemia, which may have implications for understanding the systemic nature of the infection and its impact on clinical outcomes.

**Figure 3:**
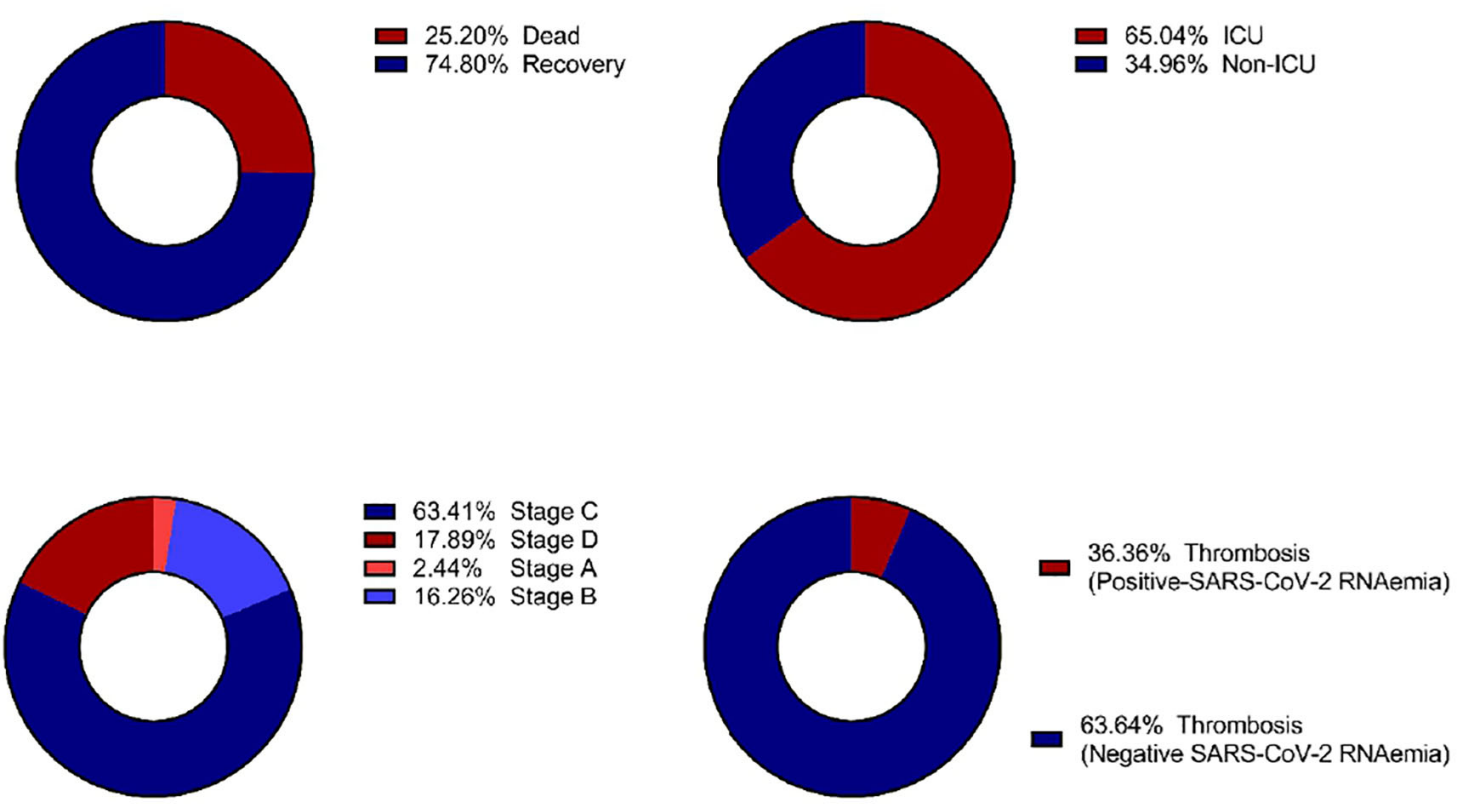
Distribution of clinical outcomes based on SARS-CoV-2 RNAemia in COVID-19 patients: **(A)** Morbidity Status, **(B)** ICU Admission, **(C)** COVID-19 Stages, and **(D)** Incidence of Thrombosis. ICU, Intensive Care Unit.

**Figure 3** illustrates the distribution of SARS-CoV-2 RNAemia in relation to ICU admissions, patient outcomes, disease stages, and the incidence of thrombosis among COVID-19 patients. Out of 123 patients with detectable SARS-CoV-2 RNA in their blood, a significant majority, 80 patients (65.04%), required ICU admission. This high percentage indicates that the presence of RNAemia may be associated with severe disease progression. The mortality rate among these patients was considerable, with 31 out of 123 (25.20%) not surviving. This suggests a potential link between RNAemia and poorer outcomes, underscoring the need for aggressive monitoring and management of patients with detectable viral RNA in their blood.

Thrombosis was diagnosed in 22 out of 446 of COVID-19 patients (4.99%). Of these, a subset of 123 patients were found to have RNAemia. Furthemore, thrombosis was diagnosed 8 out of 123 patients (6.5%) with RNAemia, indicating that while RNAemia is associated with severe disease and systemic infection, not all patients with RNAemia will develop thrombosis. This highlights the complexity of COVID-19 and the need for comprehensive monitoring of patients with RNAemia to identify those at higher risk of thrombotic events.

Interestingly, stage C of the disease exhibited the highest proportion of patients with detectable SARS-CoV-2 RNA in their blood. This finding suggests that RNAemia may be more prevalent in later stages of COVID-19, potentially serving as a marker for disease progression and severity.

### Association: differentiate between several coagulation markers in blood and nasopharyngeal samples in COVID-19 patients

Our study investigated differences in several coagulation markers between patients with positive SARS-CoV-2 RNAemia and those with SARS-CoV-2 RNA detected in the nasopharynx. The coagulation markers examined included plasminogen, α2AP, PAI-1, TAFI, tPA, aPTT, INR, platelets, fibrinogen, and D-dimer. Specifically, plasma levels of TAFI and tPA were significantly higher in patients with SARS-CoV-2 RNAemia compared to those with the virus detected in nasopharyngeal samples (P < 0.001 for both markers).

Additionally, plasma levels of fibrinogen showed a near-significant difference between SARS-CoV-2 RNA in blood samples and nasopharyngeal samples (P = 0.054), with higher fibrinogen levels observed in patients with RNAemia.

**Figure 4:**
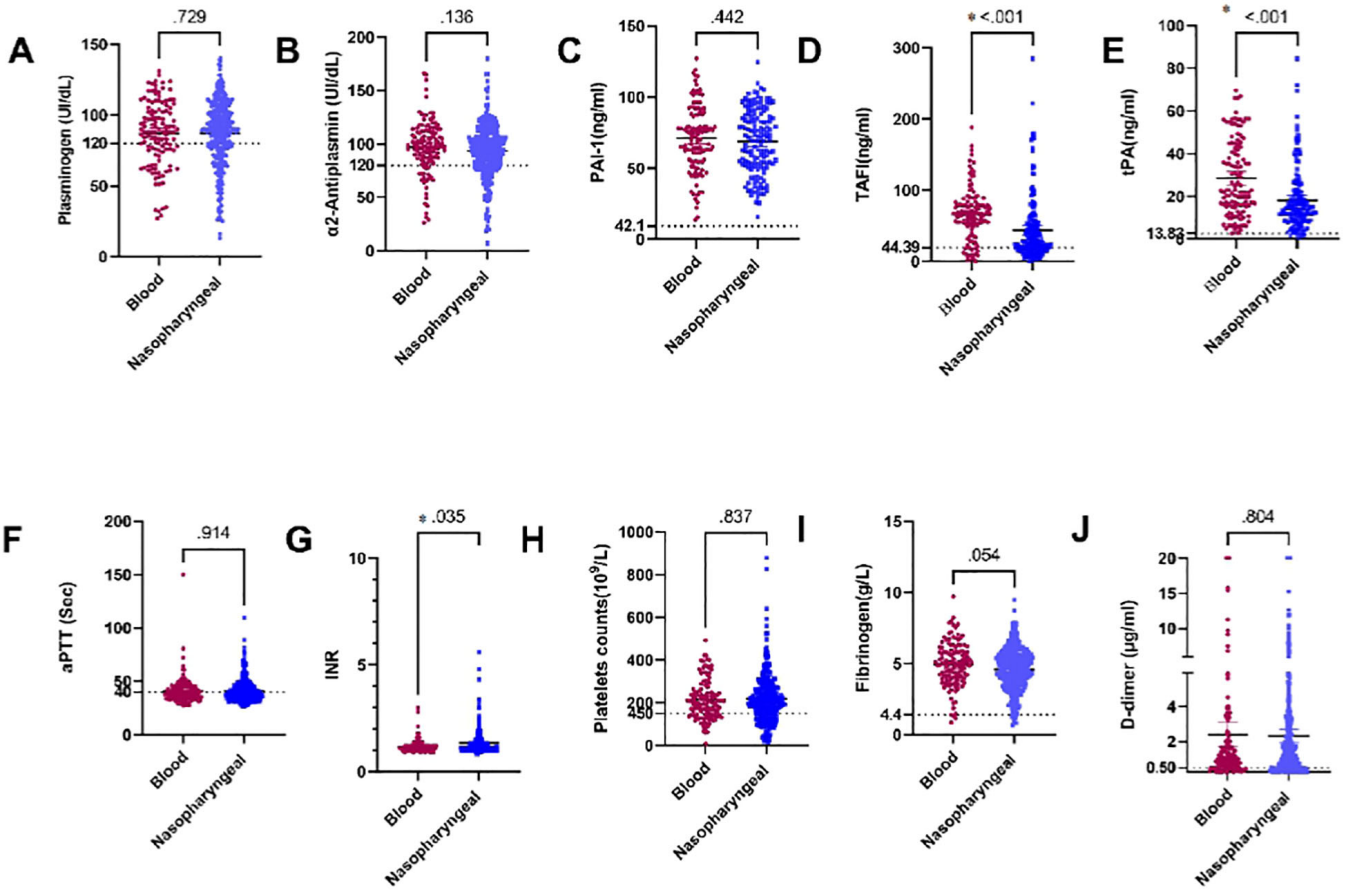
Comparison of plasma levels of coagulation factors in blood and nasopharyngeal samples in COVID-19 patients: **(A)** Plasminogen, **(B)** α2AP, **(C)** PAI-1, **(D)** TAFI, **(E)** tPA, **(F)** aPTT, **(G)** INR, **(H)** Platelets, **(I)** Fibrinogen, and **(J)** D-dimer. a2AP, Alpha2 Antiplasmin; PAI-I, Plasminogen Activator Inhibitor-1: TAFI, Thrombin Activatable Fibrinolysis Inhibitor: tPA. Tissue Plasminogen Activator: aPTT. Activated Partial Thromboplastin Time: and INR. International Normalized Ratio. Groups were compared using the Mann-Whitney U test, while normally distributed data were studied using a two- sided t-test. Data is presented as medians with 95% confidence intervals. Levels of the coagulation factors and P values are shown in each panel. The dotted lines represent the upper limit of the normal range for the plasma level of all the coagulation markers.

Furthermore, there was a significant difference in INR values when comparing SARS-CoV-2 RNA in blood samples to those in nasopharyngeal samples (P = 0.035),with higher INR values in patients with RNAemia. These findings suggest a potential association between plasma levels of certain coagulation factors and SARS-CoV-2 RNAemia, indicating that the presence of viral RNA in different sample types can impact coagulation profiles. The detailed analysis is depicted in **Figure 4**. This underscores the importance of monitoring coagulation markers in COVID-19 patients, as differences in these markers can provide insights into the disease’s impact on the coagulation system and help guide clinical management.

### Association between several coagulation markers and the N501Y, D61G, K417N and N440K mutations in the S gene in blood samples from COVID-19 patients

In our cohort, we investigated the association between several coagulation markers and specifics mutations in the S gene in blood samples from COVID-19 patients. Notably, we focus on the N501Y (19.03%), D61G (15.04%), K417N and N440K mutations had the same prevalence (9.73% in both).

**Figure 5:**
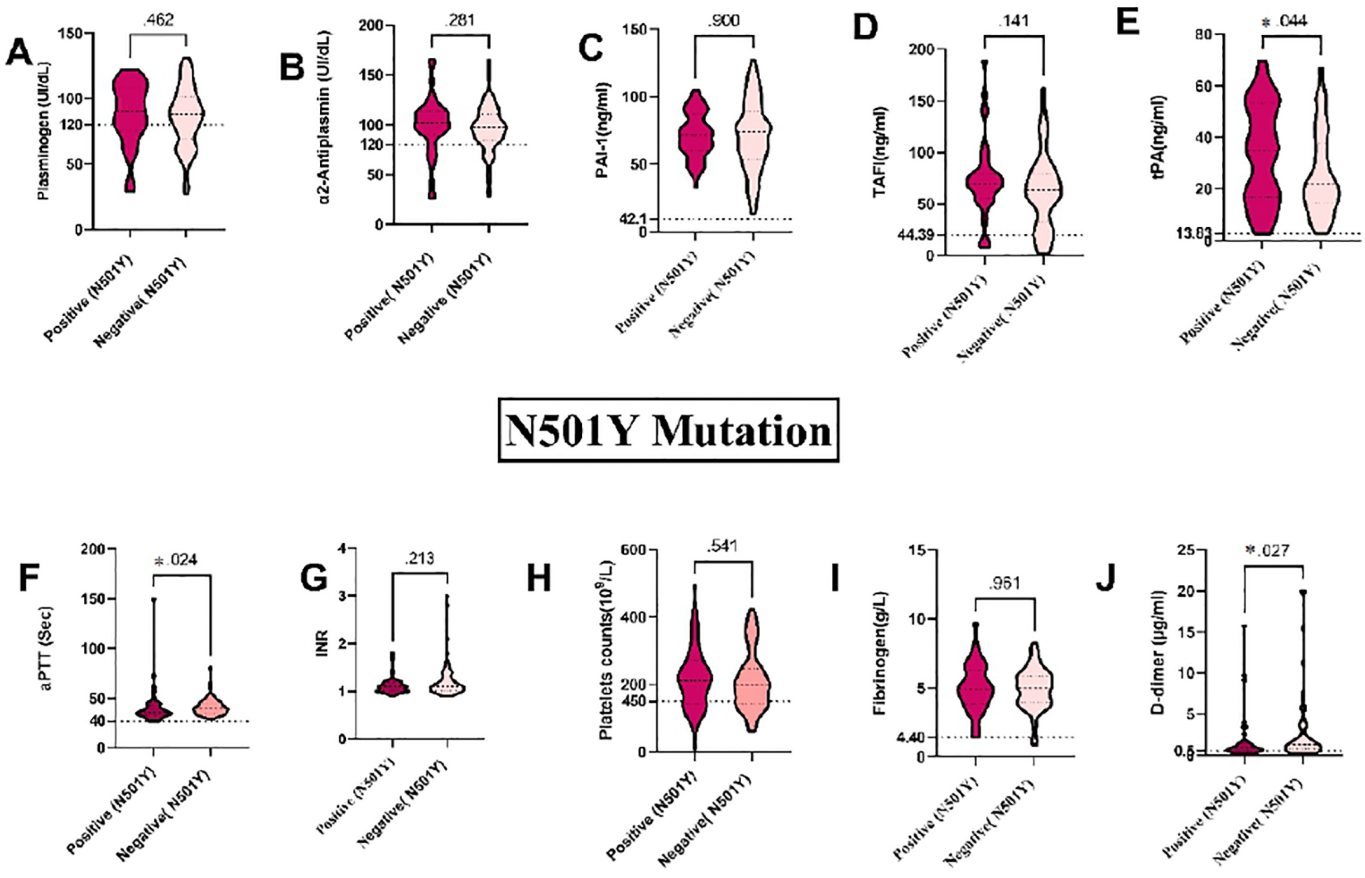
Association between coagulation markers and mutations in the S gene in blood samples from COVID-19 Patients: **(A)** Plasminogen, **(B)** α2AP, **(C)** PAI-1, **(D)** TAFI, **(E)** tPA, **(F)** aPTT, **(G)** INR, **(H)** Platelets, **(I)** Fibrinogen, and **(J)** D-dimer. a2AP, Alpha2 Antiplasmin; PAI-I, Plasminogen Activator Inhibitor-1; TAFI, Thrombin Activatable Fibrinolysis Inhibitor: IPA, Tissue Plasminogen Activator; aPTT, Activated Partial Thromboplastin Time; and INR, International Normalized Ratio. Groups were compared using the Mann-Whitney U test, while normally distributed data were studied using a two- sided t-test. Data is presented as medians with 95% confidence intervals. Levels of the coagulation factors and P values are shown in each panel. The dotted lines represent the upper limit of the normal range for the plasma level of all the coagulation markers.

Our results revealed that the N501Y mutation is the most prevalent among the SARS-CoV-2 RNA detected in blood samples, specifically within the S gene. We found a strong association between the N501Y mutation and plasma levels of tPA, aPTT, and D-dimer in blood samples from COVID-19 patients. Specifically, plasma levels of tPA and aPTT were considerably higher in patients with the N501Y mutation (P = 0.044 and P = 0.024, respectively), whereas D-dimer levels were levels were significantly lower (P = 0.027) (see **Figures 5E, F, J**)). The D61G mutation, the second most frequent mutation in our blood samples, also showed significant associations. Plasma levels of INR levels were significantly higher in individuals without the D61G mutation (P = 0.018), while platelet counts were significantly higher in those with the D61G mutation (P = 0.034). Specifically, INR levels were significantly associated with the D61G mutation (P = 0.018), and platelet counts were significantly higher in patients with the D61G mutation (P = 0.034) (see **Supplementary Figures S1(8G) and (H)**). Additionally, plasma levels of aPTT and D-dimer showed near-significant associations with the D61G mutation (P = 0.065 and P = 0.064, respectively) with higher levels observed in patients without the D61G mutation (see **Supplementary Figures S1F, IIJ**).

The K417N and N440K mutations were found in an equal number of blood samples from COVID-19 patients. Plasma levels of tPA were significantly different in patients with the N440K mutation (P = 0.023) (see **Supplementary Figure S2E**). The detection of the K417N mutation showed a near-significant association with plasma levels of tPA (P = 0.066), with higher levels observed in patients with the mutation (see **Supplementary Figures S3E**). However, there were no significant differences between the K417N and N440K mutations and other coagulation markers, including plasminogen, α2AP, PAI-1, TAFI, aPTT, INR, platelets, fibrinogen, and D-dimer (P > 0.05). These findings highlight the potential impact of the N501Y, D61G, K417N, and N440K mutations on fibrinolytic and coagulopathy changes in COVID-19 patients. Monitoring these markers can aid in risk assessment and guide clinical management strategies.

### Association between the top ten frequent mutations in thrombosis and non-thrombosis in COVID-19 patients

**Figure 6:**
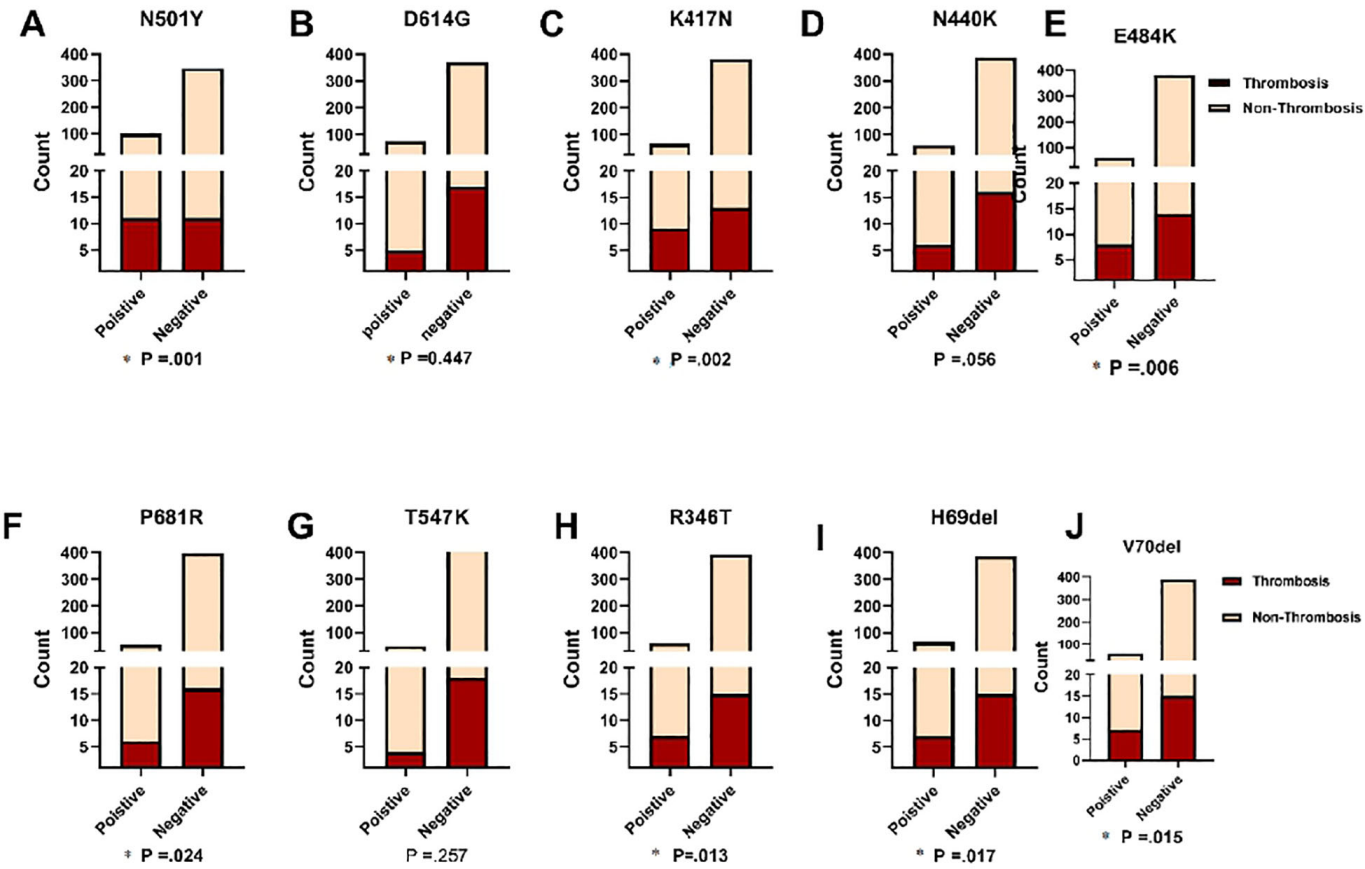
Comparison of ten significant mutations between patients with and without thrombosis, stratified by SARS-CoV-2 status. Fisher's Exact test was used and significant differences (P<0.05) are shown in bold.

**Figure 6** depicts the findings of our investigation into the association between frequent mutations and the likelihood of thrombosis development in COVID-19 patients. Our results revealed that seven out of the ten mutations examined were statistically significant predictors of thrombosis. In **Figure 6**, the terms “Positive” and “Negative” indicate the presence or absence of the N501Y mutation in the analyzed samples, respectively. Among COVID-19 patients who developed thrombosis (n = 22), the N501Y mutation was detected in 10 cases (45.5%). Furthermore, showing a significant association with thrombosis development (P = 0.001). Other mutations that were positively associated with thrombosis include K417N, E484K, P681R, R346T, H69del, and V70del (P = 0.002, P = 0.006, P = 0.024, P = 0.013, P = 0.017, and P = 0.015, respectively). However, statistical analysis showed no significant correlation between thrombotic events and the D614G or T547K mutations, with P-values of 0.447 and 0.257, respectively. Additionally, the N440K mutation showed a near-significant association with thrombosis (P = 0.056), suggesting a potential but not definitive link. These findings highlight the significance of specific mutations in predicting the risk of thrombosis in COVID-19 patients. Identifying these associations can enhance risk assessment and inform targeted clinical management strategies for patients with COVID-19.

### Association between the top ten frequent variants of thrombosis and non-thrombosis in COVID-19 patients

**Figure 7:**
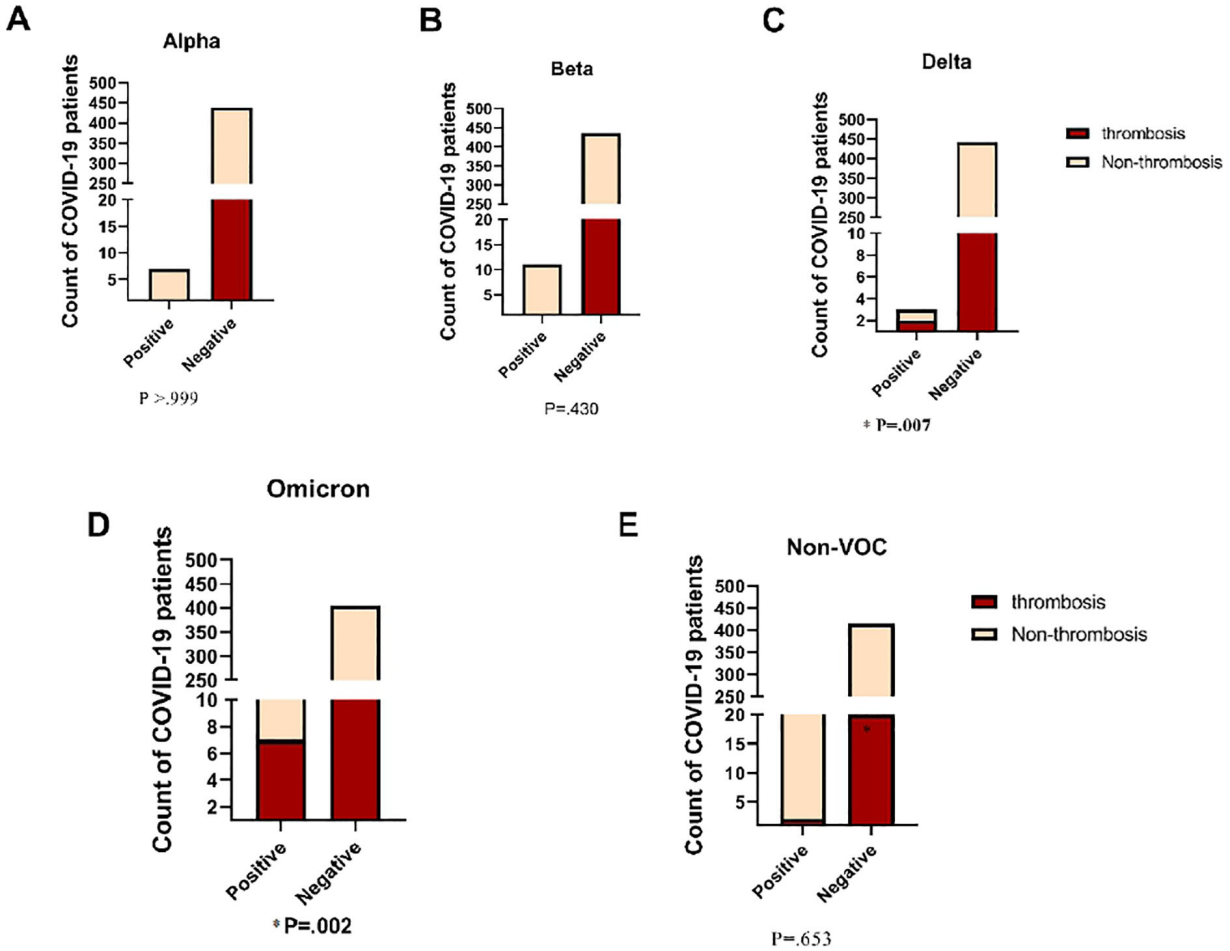
Comparison of Incidence of Thrombosis in COVID-19 Patients across Diverse SARS- CoV-2 Variants. VOC, Variant of Concern. Fisher's Exact test was used and significant differences (P<0.05) are shown in bold.

The study investigated the association between frequent variants and the likelihood of thrombosis risk in COVID-19 patients. Our results showed that the BA.1.1 strain from the Omicron variant was significantly correlated with a higher risk of thrombosis (P = 0.002). In our cohort, the Omicron variant was detected in 31.80% of COVID-19 thrombosis patients. Additionally, the Delta variant was highly significant and associated with an increased risk of developing thrombosis in COVID-19 patients (P = 0.007). However, as shown in **Figure 7**, the other variants did not show a significant difference between thrombosis and non-thrombosis cases in COVID-19 patients. These findings suggest that the BA.1.1 strain of the Omicron variant and the Delta variant are important predictors of thrombosis risk in COVID-19 patients, emphasizing the need for close monitoring and tailored clinical management for patients infected with these variants.

## Discussion

Several investigations have observed SARS-CoV-2 RNA in whole blood, serum, and plasma samples, particularly in severe COVID-19 patients (Colagrossi et al., 2021; Jacobs and Mellors, 2021). As such, accurate detection and quantification of SARS-CoV-2 RNA in the blood can serve as a valuable biomarker for assessing the clinical characteristics of COVID-19 patients (Wang et al., 2020). In our cohort, 27.58% (123 out of 446) of COVID-19 patients exhibited SARS-CoV-2 RNAemia, with RNA detected in both plasma (16.6%) and whole blood samples (6.3%). Previous studies found SARS-CoV-2 RNA in the plasma of 23% (44 out of 191) of patients and in the whole blood of 22% (2 out of 13) of patients (Ram-Mohan et al., 2020; Jacobs and Mellors, 2021). Our study included many hospitalized COVID-19 patients, whereas previous studies often relied on small sample sizes from carefully selected patients, typically those with severe or critical disease (Lax et al., 2020; Wick et al., 2022).This difference in sample size and patient selection could impact the generalizability of their findings compared to our more comprehensive study.

Our data revealed a significant correlation between SARS-CoV-2 RNAemia and disease severity, consistent with Hogan et al., 2021 (Hogan et al., 2021). Over 65% of COVID-19 patients with RNAemia were admitted to the ICU, with a 25% mortality rate. Additionally, thrombosis was observed in 36% of these patients. A meta-analysis reported that 4.28% of COVID-19 patients with RNAemia required ICU admission, with an 11% mortality rate (Johnson et al., 2021).Our investigation emphasizes the higher prevalence of RNAemia in severe cases among hospitalized COVID-19 patients, suggesting its potential as a marker for identifying those at higher risk of critical outcomes and guiding targeted therapeutic strategies.

We observed viral higher load in nasopharyngeal samples of COVID-19 patients who exhibit SARS-CoV-2 RNAemia which agrees with a systemic review reporting a high CT value in SARS-CoV-2 RNAemia cases (ranging from 33.5–44.8) (Matthews et al., 2020).Additionally, the median CT value for ICU-admitted COVID-19 patient was 37.2 (Hogan et al., 2021); suggesting the presence of SARS-CoV-2 RNA in the blood is associated with overall higher viral loads, particularly in cases with a significant lung infection burden. Consequently, SARS-CoV-2 RNAemia may be more common in severe or critical cases, underscoring its potential as a marker for severe disease progression.

Sequencing results of SARS-CoV-2 genomes from blood samples used in this study were limited, accounting for only (35%) of the total. Most of the successful sequencing outcomes were derived from plasma, rather than whole blood. The reasons behind the suboptimal sequencing results from blood samples remain poorly understood. Interestingly, even in cases where samples exhibited a high viral load, the quantified library concentrations of complementary DNA (cDNA) (using qRT-PCR) were also high. It has been shown that serum samples of COVID-19 patients contains high levels of RNases, which also correlate with severity of disease (Zechendorf et al., 2023). In addition, it has been shown that the blood contains various proteins and substances, in addition to immune reactions, which may act as inhibitory factors affecting the quality of SARS-CoV-2 viral RNA (van der Sijde et al., 2020).These factors could be responsible for the challenges encountered in sequencing blood samples.

In our cohort, patients infected with the Omicron variant predominantly presented with severe disease; all were in stage D, with most requiring ICU admission, agreeing with another study reporting a high association between the Omicron variant and severe mortality among hospitalized COVID-19 patients (Piralla et al., 2023).However, a meta-analysis of 33 studies suggested a different trend, indicating that Omicron variants generally lead to milder and less severe disease compared to the Delta variant (Hu et al., 2023).This discrepancy could be attributed to the higher likelihood of severe outcomes in unvaccinated COVID-19 patients with comorbidities who are hospitalized. Another important finding was that all Alpha and Beta patients were admitted to the ICU. A previous study reported that, in terms of COVID-19 patient outcomes under critical conditions, the Beta and Alpha variants exhibited comparable severity (Louis et al., 2022).

Studies have reported N501Y mutations in Omicron lineages (Zambrano et al., 2022). which play a crucial role in the severity of COVID-19 and contribute to immune evasion by SARS-CoV-2 (Rasheed et al., 2023). In our study, N501Y mutations were frequent among COVID-19 patients, observed in Alpha, Beta, and Omicron variants. Moreover, N501Y mutation was significantly associated with COVID-19-related mortality and higher ICU admissions. Both N501Y and D614G mutations were highly reported in stages C and D, potentially influenced by unvaccinated Alpha, Beta, and Omicron infections. Notably, patients in stage C and D, representing severe infection requiring ICU admission, exhibited a higher frequency of these mutations.

Our study highlights that the D614G mutation is associated with higher ICU admissions, contrary to earlier studies linking it to reduced hospitalization rates and improved patient survival (Esper et al., 2021). This mutation, which occurs in the Spike protein of the SARS-CoV-2 virus, has been reported in all variants. However, drawing a solid conclusion about the functional significance of D614G remains challenging. Coagulation and fibrinolysis markers were compared among SARS-CoV-2 clades in our cohort, revealing prolonged aPTT in COVID-19 patients with the N501Y mutation, associated with the Omicron variant. Additionally, increased plasma levels of D-dimer and tPA, linked to inflammation (Wannamethee et al., 2012); were recorded in these patients, potentially leading to hyperfibrinolysis. This finding supports previous research indicating differences in clotting parameters between Delta and Omicron variant groups, potentially related to acute inflammatory disorders (Tian et al., 2023).

Several studies consistently highlight an increased risk of thrombotic events among COVID-19 patients, particularly in severe cases requiring hospitalization (Lodigiani et al., 2020; Tang et al., 2020).In our study, whole genome sequencing on thrombosis patients revealed consistent mutations. Over half of venous thrombosis cases had the N501Y mutation, linked with Omicron (BA.1.1) variant. However, a patient with arterial thrombosis had the P681R mutation, usually associated with Delta variants. Interestingly, our findings contradict prior research on pulmonary embolism prevalence in Delta and Omicron variant-infected COVID-19 patients (Law et al., 2022). These differences underscore the complexity of SARS-CoV-2 mutations and their clinical impact.

An association between COVID-19-related mortality and deep vein thrombosis (DVT) and pulmonary embolism (PE) in ICU-admitted patients was identified. Among 22 studied patients, 2 (9%) developed both DVT and PE during their ICU stay, correlating with higher mortality risk. Notably, these patients carried N501Y, E484K, and K417N mutations in the S protein. Thromboembolic complications, including DVT and PE, are common causes of mortality in hospitalized COVID-19 patients, with venous thromboembolism (VTE) occurring more frequently than arterial thromboembolism (Fontelo et al., 2021; Othman et al., 2024). In agreement with our findings, an incidence of DVT ranging from 0.4% to 84% and of PE ranging from 1.0% to 40.0% of COVID-19 patients has been reported (Colagrossi et al., 2021). Previous studies have also shown that all-cause mortality of COVID-19 patients varies widely, ranging from 4.8% to 63%. Mortality specifically associated with thromboembolic complications falls within the range of 5% to 48% (Agarwal et al., 2022; Othman et al., 2024).

The SARS-CoV-2 spike protein is crucial for viral entry into host cells via the ACE2 receptor and is implicated in coagulation abnormalities and thrombotic events (Zhang et al., 2020). It can activate platelets and interact with fibrinogen, potentially causing clot formation. Free SP fragments have been detected in COVID-19 patients’ plasma. A pilot study found SP on platelets within thrombi from COVID-19 patients with acute ischemic stroke and myocardial infarction, but no SARS-CoV-2 RNA, suggesting that SP fragments alone might trigger clot formation (De Michele et al., 2022). This finding confirms the association among these mutations and venous thrombosis among COVID-19 patients (Hungaro Cunha et al., 2022).

We demonstrate that Omicron (BA.1.1) variants, including the N501Y mutation, were observed in patients with elevated PAI-1 and decreased platelets which aligns with findings of a previous study (Vallejo et al., 2022).

Significantly elevated levels of D-dimer were reported among thrombosis patients, with N501Y and D61G mutations showing close-to-significant plasma elevation compared to other mutations with normal levels. Previous research found a significant correlation between D614G mutations and high D-dimer levels among COVID-19 Omicron variant patients (Yin et al., 2022). Our study also indicates a significant correlation between elevated plasma levels of tPA and N440K and N501Y, and N501Y mutations, whereas the K417N mutation was nearly significant.

However, it’s important to note that the correlation between SARS-CoV-2 variants and evidence of thrombosis among COVID-19 patients has not been sufficiently studied. The prevalence of thrombosis among COVID-19 patients and its association with SARS-CoV-2 variants have not been thoroughly explored. Furthermore, there is a lack of research comparing specific coagulation markers, such as tPA and TAFIA, with SARS-CoV-2 variants. Further investigation is needed to better understand the thrombogenic potential across various SARS-CoV-2 strains, necessitating additional data collection and analysis.

## Conclusion

SARS-CoV-2 RNAemia is a useful tool for identifying the clinical characteristics of COVID-19 patients and predict disease severity. The N501Y, D614G, K417N, and E484K mutations are highly prevalent among COVID-19 patients. These mutations are strongly associated with COVID-19-related mortality and ICU admissions. SARS-CoV-2 has a unique mechanism for alterations in coagulation and fibrinolysis that can result in an elevated risk of thrombotic events. Elevated levels of TAFI, PAI-1, and tPA were observed in this study, suggesting their potential as prognostic markers for hypofibrinolysis.

## Data Availability

The original contributions presented in the study are included in the article/**Supplementary Material**. Further inquiries can be directed to the corresponding author.
