# Supplementary material for: SARS-COV-2 causes significant abnormalities in the fibrinolysis system of patients: correlation between viral mutations, variants and thrombosis
Source: Front Cell Infect Microbiol. 2025 Apr 15;15:1531412. doi: 10.3389/fcimb.2025.1531412 (PMC12037514; doi:10.3389/fcimb.2025.1531412)
Supplement: Supplementary file 1 [file Table1.docx]

**Supplementary Materials**


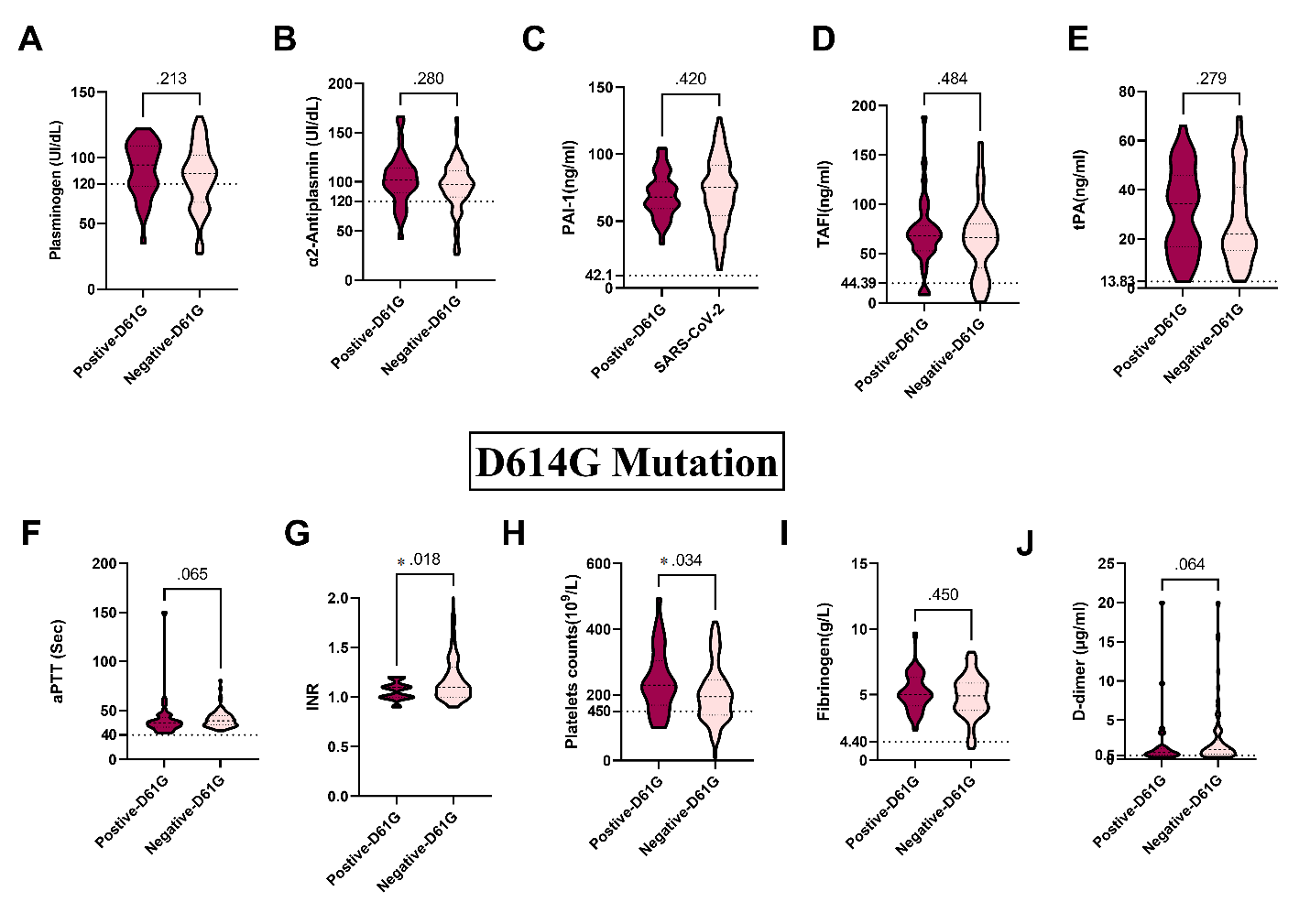


**Figure S1**. Association between Coagulation Markers and the D61G Mutation in the S gene in Blood Samples from COVID-19 Patients

**Abbreviations:** α2AP, Alpha2 Antiplasmin; PAI-I, Plasminogen Activator Inhibitor-1; TAFI, Thrombin Activatable Fibrinolysis Inhibitor; tPA, Tissue Plasminogen Activator; aPTT, Activated Partial Thromboplastin Time; and INR, International Normalized Ratio.

Groups were compared using the Mann-Whitney U test, while normally distributed data were studied using a two-sided t-test. Data is presented as medians with 95% confidence intervals. Levels of the coagulation factors and P values are shown in each panel. The dotted lines represent the upper limit of the normal range for the plasma level of all the coagulation markers.


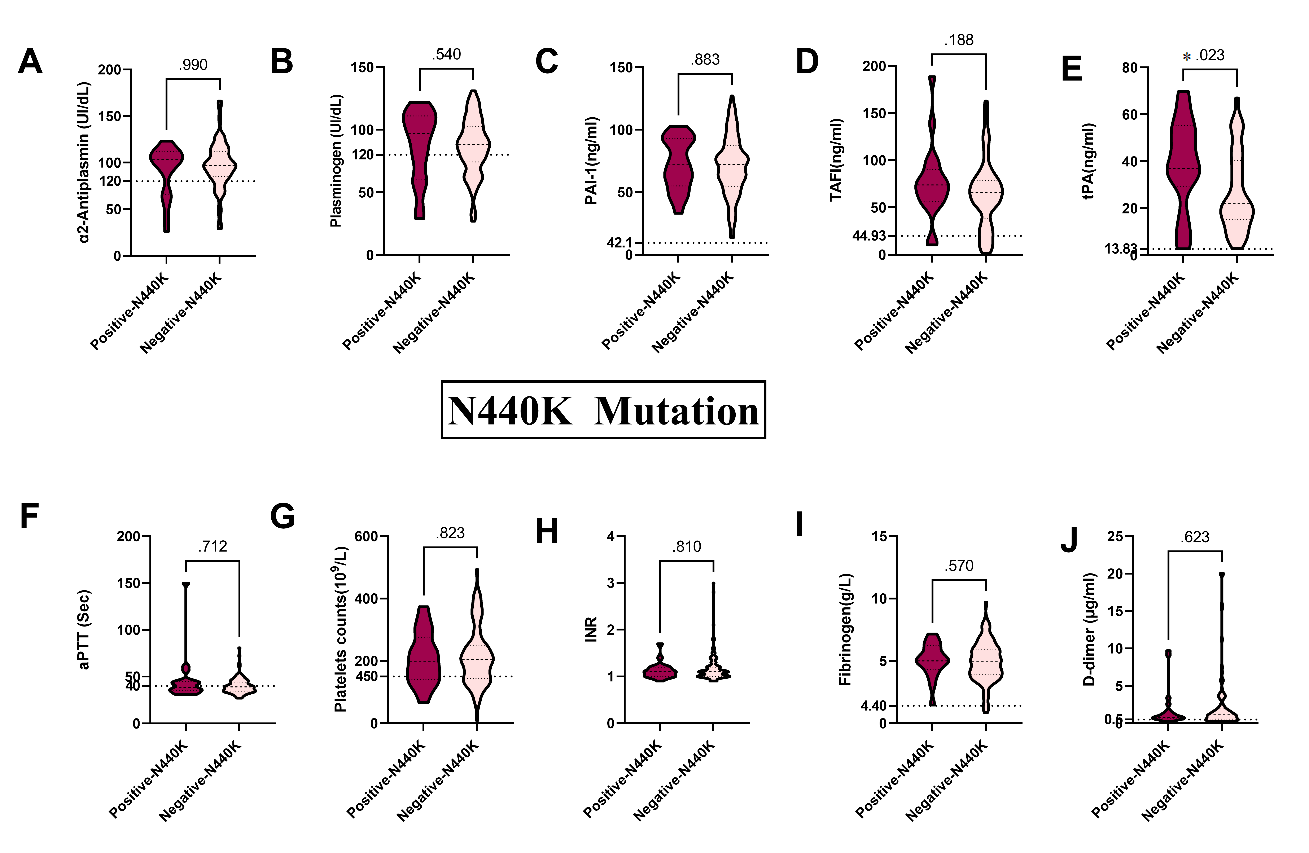


**Figure S2**. Association between Coagulation Markers and the N440K Mutation in the S gene in Blood Samples from COVID-19 Patients

**Abbreviations:** α2AP, Alpha2 Antiplasmin; PAI-I, Plasminogen Activator Inhibitor-1; TAFI, Thrombin Activatable Fibrinolysis Inhibitor; tPA, Tissue Plasminogen Activator; aPTT, Activated Partial Thromboplastin Time; and INR, International Normalized Ratio.

Groups were compared using the Mann-Whitney U test, while normally distributed data were studied using a two-sided t-test. Data is presented as medians with 95% confidence intervals. Levels of the coagulation factors and P values are shown in each panel. The dotted lines represent the upper limit of the normal range for the plasma level of all the coagulation markers.

**
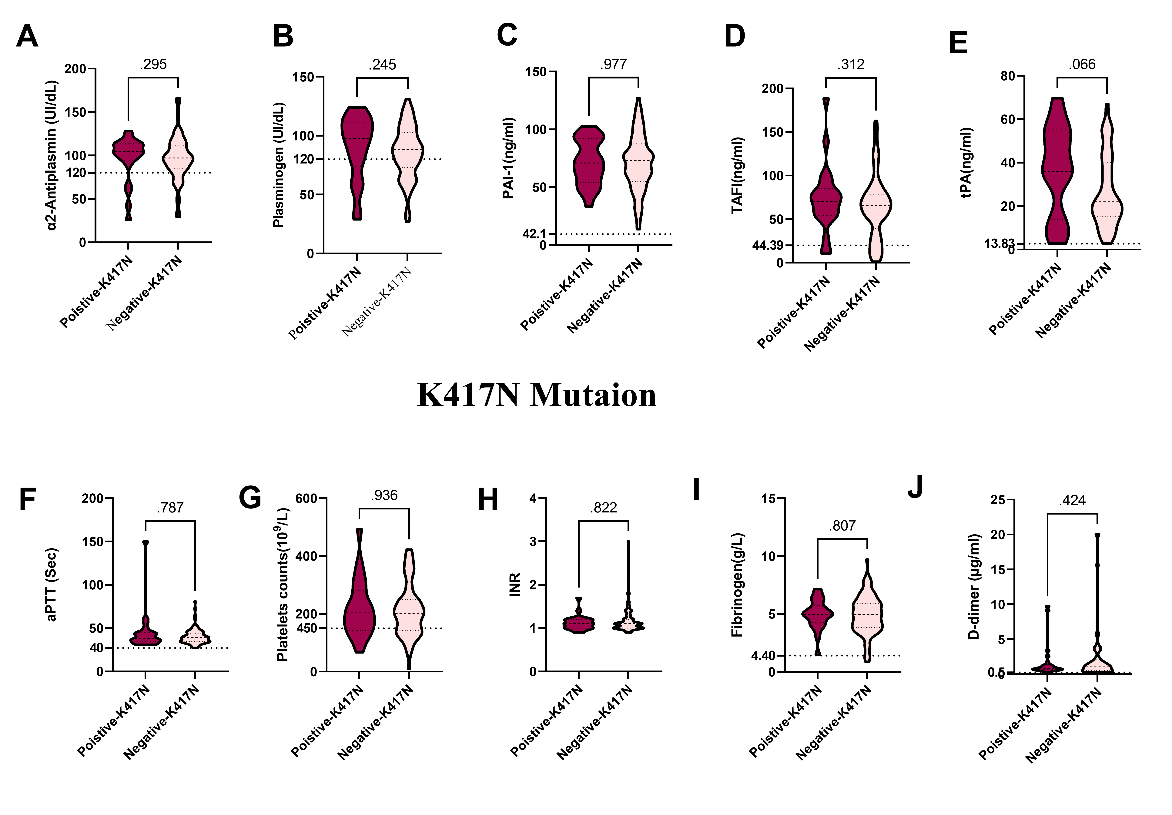
**

**Figure S3**. Association between Coagulation Markers and the K417N Mutation in the S gene in Blood Samples from COVID-19 Patients

**Abbreviations:** α2AP, Alpha2 Antiplasmin; PAI-I, Plasminogen Activator Inhibitor-1; TAFI, Thrombin Activatable Fibrinolysis Inhibitor; tPA, Tissue Plasminogen Activator; aPTT, Activated Partial Thromboplastin Time; and INR, International Normalized Ratio.

Groups were compared using the Mann-Whitney U test, while normally distributed data were studied using a two-sided t-test. Data is presented as medians with 95% confidence intervals. Levels of the coagulation factors and P values are shown in each panel. The dotted lines represent the upper limit of the normal range for the plasma level of all the coagulation markers.
